# Nucleoporin Nup358 Downregulation Tunes the Neuronal Excitability in Mouse Cortical Neurons

**DOI:** 10.3390/life13091791

**Published:** 2023-08-22

**Authors:** Vladimir A. Martínez-Rojas, Francesca Pischedda, Isabel Romero-Maldonado, Bouchra Khalaf, Giovanni Piccoli, Paolo Macchi, Carlo Musio

**Affiliations:** 1 Institute of Biophysics—IBF, National Research Council—CNR, Via Sommarive 18, 38123 Trento, Italy; vlalx.mr@gmail.com; 2 Department of Cellular, Computational and Integrative Biology—CIBIO, University of Trento, Via Sommarive 9, 38123 Trento, Italy; francesca.pischedda@unitn.it (F.P.); bouchra.khalaf@gmail.com (B.K.); giovanni.piccoli@unitn.it (G.P.); 3 Institute of Cellular Physiology, Universidad Autónoma de Mexico—UNAM, Ciudad Universitaria, Mexico City 04510, Mexico; isabel.romero@comunidad.unam.mx

**Keywords:** nucleoporins, ion channels, membrane excitability, voltage-gated sodium channels, neuronal activity, neurodegenerative disease, patch-clamp

## Abstract

Nucleoporins (NUPs) are proteins that comprise the nuclear pore complexes (NPCs). The NPC spans the nuclear envelope of a cell and provides a channel through which RNA and proteins move between the nucleus and the cytoplasm and vice versa. NUP and NPC disruptions have a great impact on the pathophysiology of neurodegenerative diseases (NDDs). Although the downregulation of Nup358 leads to a reduction in the scaffold protein ankyrin-G at the axon initial segment (AIS) of mature neurons, the function of Nup358 in the cytoplasm of neurons remains elusive. To investigate whether Nup358 plays any role in neuronal activity, we downregulated Nup358 in non-pathological mouse cortical neurons and measured their active and passive bioelectrical properties. We identified that Nup358 downregulation is able to produce significant modifications of cell-membrane excitability via voltage-gated sodium channel kinetics. Our findings suggest that Nup358 contributes to neuronal excitability through a functional stabilization of the electrical properties of the neuronal membrane. Hypotheses will be discussed regarding the alteration of this active regulation as putatively occurring in the pathophysiology of NDDs.

## 1. Introduction

The nuclear pore complex (NPC) is a large multiprotein complex of nucleoporin (NUP) proteins that mediate nucleocytoplasmic transport, genome organization, and gene expression [1,2]. An important body of studies indicates that disruptions of the NPC contribute to the pathogenesis of neurodegenerative diseases (NDDs) by triggering pathophysiological intracellular cascade effects [3,4]. NUPs were first reported to localize at the nuclear rim, but emerging evidence has revealed that some individual nucleoporins are stably expressed in the cytoplasm and/or the nucleoplasm and contribute to numerous physiological events [5,6,7,8]. Nup358, also known as Ran-binding protein 2 (RanBP2), is by far the largest nucleoporin [9] of the NPC [10]. In neurons, Nup358 is indispensable for the process of axon specification and cell polarity during neuronal differentiation in culture [11]. The conditional loss of Nup358 in Thy1^+^-motoneurons in mice causes the development of amyotrophic lateral sclerosis-like motor traits [12]. Furthermore, the loss of Nup358 in motoneurons triggers the dysregulation of neuronal–glial and chemokine signaling in sciatic nerves and somas of spinal motoneurons from mice without Nup358 [13]. Altogether, those reports underline the relevance of Nup358 in neuronal homeostasis during the development and maturation of neurons.

Recently, it has been shown that Nup358, through its N-terminal domain, is associated with the axon initial segment (AIS) in neurons in an ankyrin-G-dependent fashion [14]. Another study also confirmed that Nup358 is enriched at the AIS [15]. Stimulation through chemical depolarization of neurons results in an evident change in Nup358 subcellular distribution and/or expression, as demonstrated by (1) a reduction in the Nup358 signal at the nuclear rim, (2) more diffuse staining in the entire neuronal cell, and (3) a decrease in the total Nup358 protein expression level. Although evidence on the regulation of electrical activity exerted by nucleoporins has been reported in non-neuronal systems [16,17], the functional impact of the Nup-mediated electrophysiological activity in neuronal systems is still unexplored.

Hypothesizing that Nup358 modulates neuronal excitability, this work hence aims to determine the functional effects of Nup358 downregulation in neurons. Using whole-cell patch-clamp on dissociated cortical neurons, we show that Nup358 regulates neuron excitability by facilitating the conductance of voltage-gated sodium channels.

On the basis of functional interpretations of our results, and according to the structure/function relationship concept, we can postulate that (1) Nup358 promotes the stabilization of the membrane proteins that control neuronal firing and (2) impairments in Nup358 function might be involved in the pathophysiological mechanisms of NDDs.

## 2. Materials and Methods

### 2.1. Animals and Cell Culture

Mice (c57/bl6 strain) were kept in a normal light/dark cycle (12 h light:12 h dark) and had free access to water and food. Cortical neurons were prepared from E15 female and male embryos using a previously described method [14]. Briefly, we euthanized the pregnant mice with a carbon dioxide (CO_2_) overdose. We isolated cortices under a stereomicroscope. After mechanical dissociation, cells were counted and plated on 12 mm coverslips coated with poly-D-lysine (Sigma, St. Louis, MO, USA) according to the density of 150–200 cells/mm^2^. The cells were grown in neuronal complete medium (Neurobasal 1×, B-27 supplement 1×, 0.5 mM L-glutamine, 10 μg/mL gentamicin; B-27 supplement and Neurobasal medium were purchased at Life Technologies, Carlsbad, CA, USA) at 37 °C and 5% CO_2_ until full maturation, i.e., 14 days in vitro (DIV).

### 2.2. Constructs

Scrambled negative control shRNA (shCTRL, Origene TR30021) and a pool of four mouse gene-specific Nup358 shRNA constructs (shNup358, Origene TL501860) in pGFP-C-shLenti Vector (Origene, Rockville, MD, USA) plasmids were used for the knockdown experiments as previously described [14]. Briefly, mouse cortical neurons were transfected on DIV 5 with shRNA constructs using Lipofectamine 2000 reagent (Invitrogen, Waltham, MA, USA) following the manufacturer’s instructions and processed on DIV 14.

### 2.3. Electrophysiology

#### 2.3.1. Recordings

Patch clamp recordings, in the configuration of the whole cell, were conducted in accordance with previous reports [18,19,20]. The neurons in the culture were placed into a recording chamber and underwent constant perfusion (flow rate, 3 mL per min) with standard extracellular solution consisting of (in mM) 140 NaCl, 4 KCl, 10 HEPES, 2.0 MgCl_2_, 2.0 CaCl_2_, and 10 glucose, pH 7.4 and 290 mOsm. The micropipettes were obtained from a temperature-controlled PIP6 pipette puller (HEKA, Reutlingen, Germany) and borosilicate glass capillaries (Harvard Apparatus, Cambridge, MA, USA). The resistance of the electrodes ranged between 3 and 5 MΩ upon filling them with an intracellular solution composed of (in mM) 130 K-gluconate, 10 KCl, 0.1 CaCl_2_, 2.0 MgCl_2_, 1.1 EGTA, 10 HEPES, 2.0 Mg-ATP, and 0.2 Na-GTP, pH 7.3 (280 mOsm). The cells selected for recording were identified subsequent to the 470 nm stimulation using a fiber-coupled LED (M89L01, Thorlabs, Newton, MA, USA), and the microelectrodes were positioned, for each neuron, in a distal somatic area through a water-immersion 40× objective. Once the whole-cell configuration was established, the series resistance and membrane capacitance were electronically compensated. Experimental signals were obtained using an ELC-03XS amplifier (npi, Tamm, Germany), filtered at 2 kHz with a low-pass filter, and sampled at 10 kHz with an INT-20X interface (npi electronic, Tamm, Germany). Analogic signals were recorded using an ELC-03XS amplifier (npi, Germany) and digitized with an INT-20X interface (npi electronic, Tamm, Germany). Data were acquired with WinWCP V5.2.7 software (©John Dempster, University of Strathclyde, Glasgow, UK) and low-pass-filtered at 2 kHz. The sampling rate employed was 10 kHz. Seal stability was tracked online, incorporating the following criteria: access resistance (<20 MΩ < 20% drift), stable RMP, and holding currents <100 pA. After a brief period of stabilization, the RMP was measured in a bridge balance configuration. The ion currents and action potentials were obtained from neurons displaying an RMP that was stable and lower than −50 mV. Thereafter, the membrane’s passive properties were explored through hyperpolarizing current square pulses from 0 to −150 pA in −30 pA decreasing pulses. Still in a current-clamp configuration, a succession of depolarizing pulses required to trigger an action potential (AP) were injected from an imposed membrane potential of −70 mV, and unitary APs were evoked using 10 ms suprathreshold steps. In order to evaluate the AP frequency, we delivered long pulses (1000 milliseconds) of depolarizing current in 50 pA delta increments. Voltage-dependent ion conductances were elicited by 10 mV steps from −80 to +40 mV (500 ms) and a holding potential of −70 mV in a voltage-clamp configuration. In order to remove capacitive transients and leakage currents, both of no interest, from the macroscopic current providing only the ionic current under exam, P/4 (or P/N according to WinWCP V5.2.7 software term) leak subtraction protocol was applied online, with which capacitive and leakage currents, ideally linear and not voltage-dependent, were subtracted from the signal of interest.

#### 2.3.2. Ionic Current Dissection

The experiments designed for the isolation of sodium current were performed as previously described [20], Cs+-based intracellular solution was composed of (in mM) 130 CsCl, 10 NaCl, 2 MgCl_2_, 0.1 CaCl_2_, 1.1 EGTA, 10 HEPES, 3 MgATP, 0.3 GTP, and pH 7.2 adjusted with CsOH, and 4-amino pyridine, 4-AP (20 μM) and tetraethylammonium, TEA (200 μM) were also added to the bath solution. The voltage dependency of activation was computed through a stepwise procedure starting from a holding potential of −70 mV and with increasing voltage steps of +5 mV across a range of potentials from −80 to +40 mV. The voltage dependency of inactivation was explored using a two-step procedure, with an initial conditioning pulse lasting 40 ms delivered from a holding potential of −70 mV to a series of potentials from −80 to 0 mV in 10 mV increments, succeeded by a test pulse to +10 mV. To analyze the recovery from fast inactivation, we employed a multiple-pulse procedure executed with a conditioning depolarization from a holding potential of −70 to −15 mV (10 ms); then, a subsequent test pulse at the equal depolarizing pulse value was delivered beforehand to evoke the Na^+^ current fraction. The time between conditioning and test pulses (Δ) was modified throughout 2–18 ms, in 2 ms augmentations, to analyze the rate of recovery [21].

### 2.4. Data Analysis

Mean ± SEM values are provided for the data, with n representing the sample size of cells recorded. The AP waveform kinetics were calculated by differentiating the spike voltage with respect to time (dV/dt). Plane phase graphs were created by plotting the first derivative of the membrane potential (in mV/ms) against the membrane voltage. The threshold was established based on the membrane potential, where dV/dt exhibited an abrupt increase. The current density was estimated by dividing the peak current values by the total membrane surface area obtained from whole-cell capacitance determination. Conductance (G) was calculated through the equation G(V_m_) = I(V_m_)/(V_m_ − E_rev_), where I(V_m_) is the current measured at the used membrane potential and V_m_, and E_rev_ is the computed Nernst potential. The maximum conductivity (G_max_) of every cell was used for normalization. To determine a measure of gating conductance, peak currents were adjusted using a Boltzmann distribution with the form G = G_0_ + (G_max_ − G_0_)/1 + exp[(V_1/2_ − V)/k], where G refers to the conductance that varies with voltage, while G_0_ represents the voltage-independent baseline conductance. V_1/2_ is the voltage of half-maximal activation, while k denotes the slope factor employed to calculate the activation kinetics [20]. The inactivation time constants were obtained by plotting the peak current amplitude during each test pulse normalized to the current amplitude of the first test pulse and graphed as a function of the pre-pulse potential. Last, the Boltzmann equation was used to fit the values of the voltage dependency of inactivation. The time constant of the recovery from inactivation was obtained by plotting the ratio I_test_/I_conditioning_ against the interval time (Δt) and the data were fitted with a single exponential equation. In order to perform data analysis, curve fitting, and plotting, we used the WinWCP, OriginPro 8.1 (OriginLab, Northampton, MA, USA), and Prism 8 (GraphPad, Boston, MA, USA) software packages, respectively. The Kolmogorov–Smirnov test was employed to test data normality. An unpaired Student’s t was employed when comparing two groups; in all analyses, a threshold for statistical significance was set at *p* < 0.05.

## 3. Results

**Figure 1:**
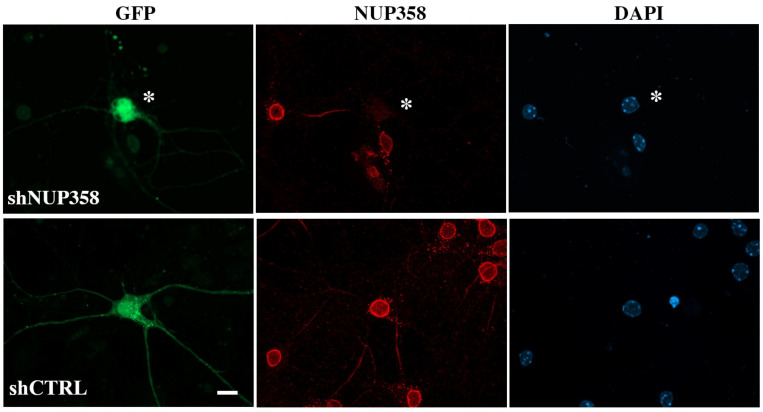
Downregulation of Nup358 expression in cortical neurons. Cortical neurons transfected at 5 DIV, with shRNA constructs co-expressing GFP to downregulate Nup358 (shNup358). As a control, neurons were transfected with a non-targeted shRNA construct (shCTRL). Immunostaining was conducted for Nup358 (red), and the nuclei are labeled with DAPI (blue). Asterisks are used to mark the transfected neuronal cells (green) with a substantial decrease in the Nup358 signal compared to the control untransfected cells. Scale bar: 10 µm.

We first downregulated Nup358 using RNAi as previously described [14]. Neurons were transfected either with an shNup358 or with a non-targeted shCTRL construct as a negative control. The efficiency of the shRNAs in eliminating the expression of Nup358 was evaluated via immunostaining (Figure 1). Transfected neurons showed a significant reduction in Nup358 signal compared to shCTRL transfected neurons.

**Figure 2:**
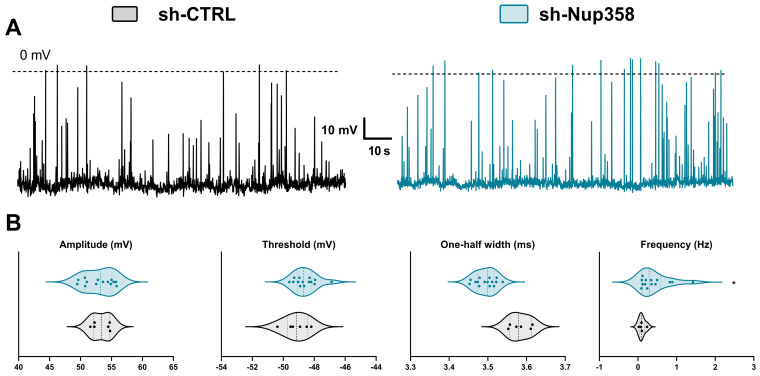
The effect of Nup358 downregulation on the spontaneous activity of cultured mouse cortical neurons. (**A**) Tracks of membrane potential changes recorded in gap-free mode in the control scrambled-treated (**left**) and shNup358-treated (**right**) neurons. (**B**) Different properties of the resting membrane potentials are computed from the traces shown in (**A**). Each dot in the graphs represents an individual cell, and the dashed line in the violin plots is the median; the left and right dashed lines are the upper and lower quartiles, respectively. *n* = 14. * *p* < 0.05, Student’s unpaired *t*-test.

We subsequently examined the effect of Nup358 downregulation on the spontaneous activity of the cultured mouse cortical neurons at 12 days in vitro (DIV12). The transfected neurons were identified through the presence of the reporter protein, GFP, and only those exhibiting positive expression were chosen for whole-cell patch-clamp recordings. At their respective physiological resting membrane potential (RMP), we tracked the intrinsic neuronal activity and found no significant differences in the RMP between the neurons with induced downregulation of Nup358 (56.85 ± 1.52 mV) and those treated with scrambled shRNA (57.18 ± 1.17 mV) (Figure 2A). While we did not detect differences in the amplitude, threshold, or the one-half width of the action potential (Figure 2B), the frequency of membrane potentials was significantly higher in the shNup358-treated neurons (0.42 ± 0.10 Hz) than in the control neurons (0.10 ± 0.03 Hz; here and throughout the text, values are mean ± SEM).

Next, we assessed whether Nup358 downregulation alters the active neuronal membrane properties. We injected neurons with 1000 ms long hyperpolarizing/depolarizing current pulses of increasing amplitude and recorded variations of membrane potentials from the membrane resting potential.

**Figure 3:**
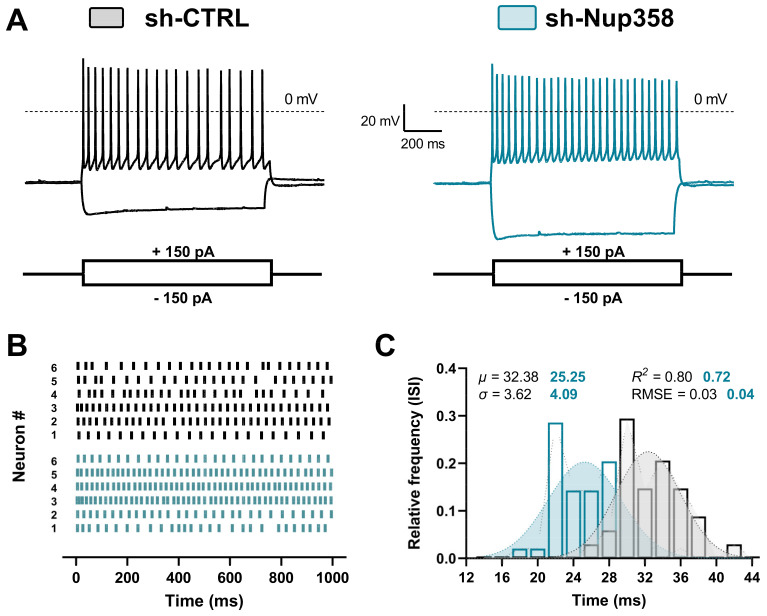
The downregulation of Nup358 increases the firing frequency of cortical neurons. (**A**) Representative recordings of membrane potentials in scrambled and shNup358-treated neurons in response to 1000 ms long whole-cell current injections. (**B**) Raster plot showing the temporal locations of action potential firing over current injection trials. Each bar in the raster indicates one action potential, and each row represents an independently recorded neuron. (**C**) Relative frequency distributions of the mean Inter Spike Interval (ISI) computed from the neurons assessed in (**B**). *n* = 6 + 6. µ, Gaussian fit mean; σ, Gaussian fit S.D.; RMSE, root-mean-square deviation.

The injection of a 150 pA current evoked a tonic firing in neurons from both experimental groups (Figure 3A); however, the number of spikes was significantly higher in the shNup358-treated neurons. We performed raster plot and frequency analyses to estimate the shNup358-mediated rise in the action potential tonic firing.

Compared to the control group, the downregulation of Nup358 resulted in an increase in the number of spikes per individual neuron and a leftward shift in the InterSpike Interval (ISI) temporal window, indicating prompter excitability of the neurons (Figure 3B,C). These results demonstrate that Nup358 plays a critical role in regulating the resting membrane potential and the frequency of action potential firing of neurons.

**Figure 4:**
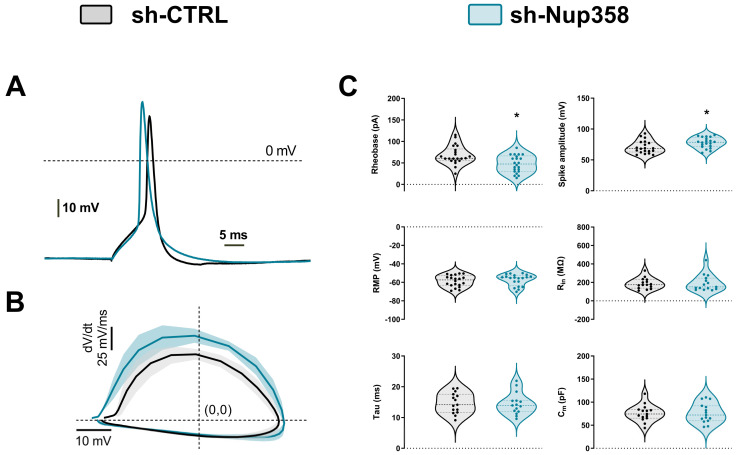
Downregulation of Nup358 alters the intrinsic excitability of neurons. (**A**) Representative recordings of single APs elicited with a 10-millisecond current pulse delivered to control or shNup358-silenced neurons. (**B**) Phase plane plots (dV/dt versus membrane voltage) from averaged single APs. The central line represents the mean, and the SEM is shown as a faded area. (**C**) Violin graph comparing the rheobase and spike amplitude (as well as passive membrane properties) in Nup358-downregulated neurons. Graphs of grouped data show individual cells and mean ± SEM from cortical neurons pooled from at least four independent brain dissections for each condition. * *p* < 0.05, Student’s unpaired *t*-test. *n* = 19. RMP, resting membrane potential; R_in_, input resistance; Tau, membrane time constant; C_m_, membrane capacitance.

Further, we analyzed whether Nup358 downregulation impacts the biophysical properties of the single action potential. Figure 4A illustrates two representative traces of a single action potential reaching the firing threshold. These traces were obtained by injecting 10 ms of somatic depolarizing current into either the control or the shNup358 neurons. To assess the detailed action potential kinetics in detail, we assembled phase plots from overlaid means of single action potentials.

We examined changes in different parameters of individual spikes’ waveforms (Figure 4C). The amplitude of the AP in shNup358-silenced neurons was higher and significantly different from control neurons, with a computed increase of 7.3 ± 1.90 mV. The minimum amount of current required to evoke an AP (rheobase) was 47.50 ± 4.24 mV smaller in Nup358-silenced neurons compared to control neurons, which had 68.00 ± 4.58 mV (Figure 4B). No such differences were observed between the mean of resting membrane potential, input resistance, capacitance, or membrane time constant (tau) membrane properties.

Overall, Nup35-silenced neurons exhibited a faster firing and larger amplitude of action potentials. These results thus suggest that Nup358 modulates the active membrane properties, that is, the input–output relationships and the repetitive AP firing of individual neurons.

Since the AIS—by defining a specific neuronal compartment with the lowest firing threshold—serves as the site for action potential (AP) initiation, we investigated the potential impact of ionic conductances involved in AP generation, considering that Nup358 interacts with the scaffold protein Ankyrin-G, which plays a crucial role in the assembling the axon initial segment (AIS) and the recruiting of other molecules like voltage-dependent sodium channels (Na_v_) [22]. Hence, using the voltage-clamp configuration and clamping neurons V_m_ at −70 mV, we examined whether the voltage-gated total ionic currents underlie the increase in neuronal activity of shNup358-silenced cortical neurons.

**Figure 5:**
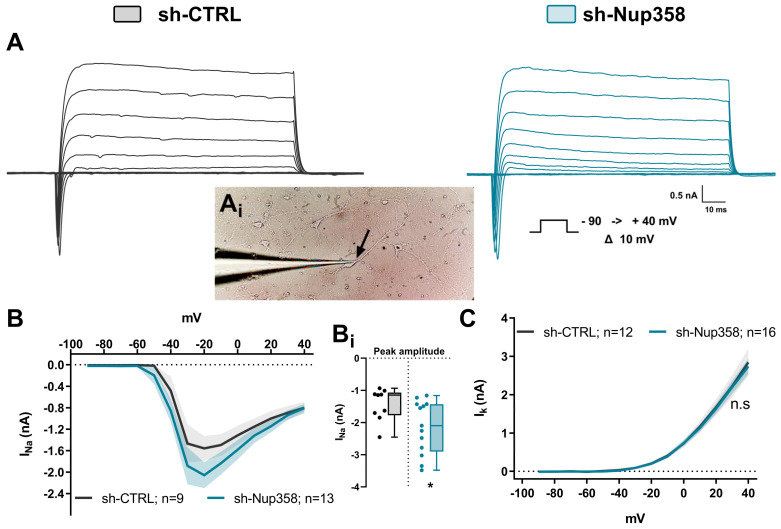
Inward total ionic current increase in shNup358-treated neurons. (**A**) Ionic current traces obtained from voltage-clamped cortical neurons. The neurons were held at −70 mV, and then voltage steps of 100 milliseconds from −90 to +40 mV were applied in 10 mV increments. (Inset **A_i_**) Exemplification of cultured cortical neurons from mice at 12DIV sealed with a glass micropipette (**left**). All the whole-cell patch clamp recordings in this work were performed in the optically identified AIS surrounding area (black arrow). (**B**) Current–voltage (I–V) relationships of the inward deflection (peak) of the traces are depicted in (**A**), left. (Inset **B_i_**) Analysis of the mean differences in maximum current amplitude at −20 mV. (**C**) Current–voltage relationships of the outward deflection (steady state) of the traces depicted in (**A**) right. The total number of assessed neurons is shown in each panel. * *p* < 0.05, Student’s unpaired *t*-test; n.s., not significant.

The step stimulation elicited voltage-gated macroscopic currents composed of Na^+^-associated inward and K^+^-associated outward currents (Figure 5A). To record the ionic responses belonging to the AIS, we placed the patch pipette in a position distal to the somatic compartment and delivered the voltage pulses (Figure 5(A_i_)). The analysis of the steady-state (outward) or peak (inward) total currents at the maximum amplitude revealed an increase of 39.05 ± 3.56% in the amplitude of the inward current in shNup358-treated cells as compared to control neurons (Figure 5(B,B_i_)). We did not observe modifications between the control and Nup358-silenced neurons when the potassium-associated outward component was compared (Figure 5C).

**Figure 6:**
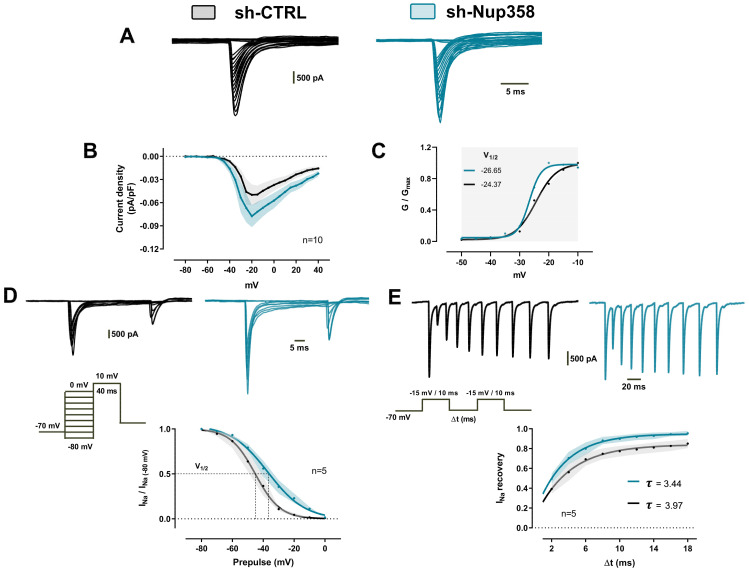
Nup358-dependent modifications of sodium currents’ biophysical properties. (**A**) Cs-based isolation of voltage-gated sodium currents (I_Na_) in response to voltage steps between −80 and +40 mV. (**B**) I-V plot of normalized peak I_Na_ currents plotted with current density as a function of test voltage. (**C**) Normalized mean conductance versus voltage plot of I_Na_ activation of the data in (**B**). The line represents the average Boltzmann fit to data. (**D**) Representative currents of I_Na_ steady-state inactivation (inset, voltage protocol). Currents were normalized to the current elicited from a holding potential of −80 mV and used to construct the steady-state inactivation. Plotted points represent the mean of the normalized currents and are fitted with a Boltzmann function. (**E**) Representative I_Na_ current traces were obtained using the protocol shown in the inset to study the recovery time course from fast inactivation. The plot represents the current recovery as a function of the interstimulus interval (Δt). Recoveries were fitted with one exponential.

Based on the previous finding, we sought to dissect sodium-associated inward currents using pharmacological and ionic substitution procedures (Figure 6A). The amplitude of the Na^+^ currents was increased in shNup358-treated neurons at the maximum peak of the curve (−20 mV). We measured the current density using the ratio of peak current amplitude to the cell membrane capacitance (pA/pF). This formal analysis computed an increase of 56% ± 3.2 of the normalized current density obtained from silenced Nup358 neurons when compared to control neurons (Figure 6B). To measure the voltage dependence of sodium conductance, the IV curve data from Figure 6B were then employed to construct a conductance/voltage (GV) plot. Then, the normalized (G/G_max_) GV relationships were fit using a single Boltzmann function (see methods for details). From this analysis, we obtained the potential of the half-maximal activation (V_1/2_) and the slope value (*K*). In control neurons, the calculated values for V_1/2_ and *K* were −24.37 ± 0.68 mV and 3.49 ± 0.61, respectively. Interestingly, in neurons treated with shNup358, the V_1/2_ showed a slight hyperpolarization of −26.65 ± 0.35 mV, and the *K* value decreased to 1.96 ± 0.27 (Figure 6C). At this point, the results clearly indicate that Nup358 is a key modulator of sodium conductance in the AIS.

To gain more insight into the biophysical mechanisms by which the Na^+^ conductances could be altered in the absence of Nup358, we measured steady-steady fast inactivation of Na^+^ currents elicited with voltage commands (pre-pulse) from −80 to 0 mV (10 mV step increase, 40 ms) in cortical neurons held at −70 mV. This allows the channels to undergo fast inactivation without permitting the channels to slowly inactivate and then to depolarize to a test pulse (10 mV) to measure the fraction of channels that did not fast inactivate. As Figure 6D shows, Nup358 silencing induced a positive shift in the I_Na+_ (voltage-gated sodium current) inactivation curve (V_1/2_ = −36.97 ± 0.68 mV and *K* factor = −12.04 ± 1.15) compared with the control ones (V_1/2_ = −45.19 ± 0.92 mV and K factor = −8.07 ± 0.85). Finally, we used a multiple-pulse protocol with recovery durations between 2 ms and 18 ms to further characterize the recovery from fast inactivation properties (inset, Figure 6D). Compared to the control, Na^+^ channels from Nup358-silenced neurons recovered rapidly from inactivation when held at negative voltages between pulses. The computed recovery time constant in shNup358 was 3.44 ms, a value considerably lower than the 3.97 ms estimated from scrambled neurons (Figure 6E). Taken together, the data obtained in this work strongly suggest that Nup358 promotes a fine-tuned control of sodium conductance in the AIS.

## 4. Discussion

### 4.1. Nup358 Downregulation and the Neuronal Excitability Alteration

Our results provide the first evidence that the downregulation of Nup358 results in increased neuronal excitability associated with changes in the biophysics of voltage-gated sodium channels. Previously, the Macchi group highlighted the unique localization of Nup358 protein expression at the AIS of cultured neurons and its relevance to neuronal activity [14]. Here, we examine in depth the electrophysiological implications of reduced Nup358 expression in cortical neurons. It is well known that the AIS has a pivotal role in triggering APs and determining neuronal output. The biophysical properties of the AIS are primarily determined by the molecular assembly and the concentration of voltage-gated channels’ proteins in this specialized region. The increased excitability observed with Nup358 silencing is consistent with the hypothesis that nucleoporin regulates the abundance, membrane distribution, or activity of some proteins, such as voltage-gated ion channels.

Previous studies have examined the role of individual Nups in the modulation of voltage-gated ion channels in cardiac electrophysiology. For instance, Nup50 induces an increase in the transcription and translation of the *Kcna4* gene, which encodes a K^+^ voltage-gated channel (Kv1.4) of shaker-related subfamily member 4, which is essential for repolarization in cardiomyocytes [16]. Nup107 also regulates cardiac bioelectricity by controlling the nucleocytoplasmic trafficking of a sodium channel mRNA (*Scn5a*) [17,23]. As is well known, both sodium and potassium voltage-gated ion channels are fundamental to the initiation and the conduction of APs in excitable cells, respectively [24]. These studies thus underscore the role of individual Nups in regulating the activity of excitable cells by modulating the expression of voltage-gated channels. In a similar manner, Nup358 might modulate the expression of proteins either through direct/indirect transcriptional control of the genes encoding the ion channel subunits or by changing the expression of genes encoding enzymes altering ion channel activity through post-translational modifications.

An emerging number of reports indicate the role of nucleoporins in the homeostasis impairment of neuronal cells and thus having ramifications in neurodegenerative disease. For instance, the alteration of the Nup153 expression leads to the functional impairment of hippocampal neural stem cells in the AD mouse model [25]. Moreover, Ranbp2/Nup358 has been implicated in the regulation of the manifestations associated with Parkinson’s disease [26]. The conditional ablation of Nup358 in mouse motor neurons causes disruptions in cellular functions and the development of amyotrophic lateral sclerosis (ALS)-like syndromes [12]. Furthermore, an in vitro/in vivo study reports that Nup358 contributes to neuronal activity and that its downregulation disrupts the functional properties of neurons regardless of the molecular domain of expression [27].

Using patch-clamp recordings, we observed a marked increase in evoked and spontaneous APs discharge upon Nup358 silencing in neurons. This hyperexcitability of neurons due to a reduction in Nup358 expression is the first demonstration linking a formerly recognized nuclear protein with an electrophysiological event. We found an increment in the amplitude of I_Na_ from Nup358-silenced neurons. Guan et al. [17] likewise found a rise in the amplitude of Nav1.5 channel currents in cardiac myocytes, but that increment was the cellular result of Nup107 overexpression.

We further explored the biophysical mechanisms of voltage-gated sodium currents. To note, we did not find changes in the voltage-gated potassium currents between control and Nup358-silenced cortical neurons, as reported for another nucleoporin family [16]. Since the activity-dependent alterations of Na^+^ channels would be predicted to have functional effects on neuronal excitability [28], we explored the kinetic properties of the activation/inactivation of voltage-gated Na^+^ channels in cortical neurons subjected to Nup358 downregulation. The kinetics of the voltage dependency of activation and the steady state from the fast inactivation of voltage-dependent Na^+^ channels were modified in Nup358-silenced neurons. In accordance with this, others have reported a functional alteration of the voltage-dependent Na^+^ channel kinetics in neurons from the central nervous system [29,30]. Collectively, these studies demonstrate that alterations induced by Nup358 in Na^+^ channel functions may underlie cortical neuron hyperexcitability. Among the alterations, we report (1) the presence of increased inward current, (2) a shift in steady-state activation or inactivation, and (3) a modification of channel recovery from fast inactivation). Moreover, we have noted that Nup358 is a fast-reactive protein responding to neuronal insults [13]. The chemical depolarization of cortical neurons with KCl modified the subcellular distribution and protein expression of Nup358 [13].

Taken together, our data show that, besides its role in nucleocytoplasmic transport, Nup358 markedly contributes to neuronal activity by modulating sodium currents. However, we cannot exclude those additional molecular mechanisms, such as anchoring proteins like AnkG, which could contribute to neuronal plasticity and in turn exert a fine-tuned regulation of Na^+^ channels kinetics. Moreover, according to our functional data, the involvement of other ionic channels/conductance (e.g., potassium, calcium, etc.) also in Nup-regulated neuronal cells, as reported for K^+^ ionic channel in cardiomyocytes [16,17], cannot be excluded a priori and could be worth investigating. Nevertheless, the functional implication of Nup358 downregulation has been limited to single-cell excitability. Finally, the study of the role of endogenously expressed Nup358 in regulating or preventing cell hyperexcitability would be of great translational interest.

### 4.2. Nup358 Downregulation: A Promising Window into Ion Channel Pathophysiology in Neurodegenerative Diseases?

On the basis of our results, it would be interesting to examine the effect of the conditional depletion of Nup358 on the final output of a neuronal network to assess the potential contribution (i.e., the active role) to the pathogenesis and/or pathophysiology of NDDs.

Nowadays, it is certain that the intracellular machinery constituted by Nuclear-Pore-Complex–Nucleoporins–Nucleocytoplasmic Transport (NCP-Nup-NCT) is emerging as a key player in the cell alterations as well as in the pathophysiology of NDDs [2,3,4,31]. Although the cause–effect relationship between NCT defects and NDD pathogenesis has still not been fully assessed, several lines of evidence lead to the conclusion that they are undoubtedly associated with many neurodegenerative disorders [23,31]. The link between the alterations of the aforementioned machinery and the pathophysiology of NDDs has been demonstrated at molecular and cellular levels, e.g., in AD (Alzheimer’s disease) [25,32]; ALS (amyotrophic lateral sclerosis) [33,34], encephalopathy [35], and, very recently, primary tauopathies [36].

Nevertheless, how the NPC-Nup-NCT machinery alterations influence neuronal excitability remains less understood. Accordingly, our electrophysiological results obtained in non-pathological cell models, to our knowledge, are the first ones in the field to demonstrate an active role of Nup358 in modulating the neuronal activity in native mouse cortical neurons, mainly through the gating of voltage-gated sodium channels.

Consequently, we are confident in hypothesizing and verifying that our results obtained in native/NUP downregulated cortical cells could be a proof of concept to verify if common biophysical and pathophysiological mechanisms related to the altered neuronal excitability via Nup358 downregulation could also occur in NDDs affected neuronal cell models.

## 5. Conclusions

Taken together, our data show that Nup expression tunes the cell membrane excitability in non-pathological cortical cells mainly acting on the gating of voltage-gated Na channels.

Although they were obtained on non-pathological cell models, we hope that our findings might constitute seminal results towards the functional study of Nup-related defects, which will need future experiments. Succeeding in this, the electrical correlate of altered neuron membrane excitability due to ion channel dysregulation, as already effectively proposed as a putative target/marker of the disease [18,19,20,37], could be extended at the NPC-Nup-NCT machinery level. Accordingly, our future goals will comprise the developing of ion channel-targeted neurotherapies based on the Nup pathogenetic cascade, according to modern pharmacogenetics and personalized pharmacology guidelines [38,39] established for neurological disorders.

## Data Availability

Relevant data are contained within the article.
